# A *crystal*-clear zebrafish for *in vivo* imaging

**DOI:** 10.1038/srep29490

**Published:** 2016-07-06

**Authors:** Paride Antinucci, Robert Hindges

**Affiliations:** 1 MRC Centre for Developmental Neurobiology, King’s College London, Guy’s Campus, London SE1 1UL, UK

## Abstract

The larval zebrafish (*Danio rerio*) is an excellent vertebrate model for *in vivo* imaging of biological phenomena at subcellular, cellular and systems levels. However, the optical accessibility of highly pigmented tissues, like the eyes, is limited even in this animal model. Typical strategies to improve the transparency of zebrafish larvae require the use of either highly toxic chemical compounds (e.g. 1-phenyl-2-thiourea, PTU) or pigmentation mutant strains (e.g. *casper* mutant). To date none of these strategies produce normally behaving larvae that are transparent in both the body and the eyes. Here we present *crystal*, an optically clear zebrafish mutant obtained by combining different viable mutations affecting skin pigmentation. Compared to the previously described combinatorial mutant *casper*, the *crystal* mutant lacks pigmentation also in the retinal pigment epithelium, therefore enabling optical access to the eyes. Unlike PTU-treated animals, *crystal* larvae are able to perform visually guided behaviours, such as the optomotor response, as efficiently as wild type larvae. To validate the *in vivo* application of *crystal* larvae, we performed whole-brain light-sheet imaging and two-photon calcium imaging of neural activity in the retina. In conclusion, this novel combinatorial pigmentation mutant represents an ideal vertebrate tool for completely unobstructed structural and functional *in vivo* investigations of biological processes, particularly when imaging tissues inside or between the eyes.

In order to understand complex biological phenomena, structural and functional information has to be extracted from intact animal systems at different spatial scales. Optical transparency of animals is a highly desirable feature to study these biological processes *in vivo*. Pigment molecules, such as melanin, haemoglobin and myoglobin, are the main sources of visible light absorption in biological tissues^1^,^2^. Lipids and collagen, on the other hand, constitute the primary molecules responsible for light scattering. Recently, several tissue clearing techniques have been developed to reduce light scattering in fixed biological tissues by selectively removing lipids in a non-destructive way^3^. However, the only molecules that can be removed from living systems without dramatically impairing their viability are pigment molecules localised in defined tissues, such as melanin present in the skin. Here, we used a combinatorial genetic approach to generate a viable, fully transparent zebrafish mutant, which we name *crystal*, lacking the vast majority of skin pigmentation. Compared to the previously described combinatorial pigmentation mutant *casper* that has pigmented eyes^4^, the *crystal* mutant constitutes a significant improvement for *in vivo* imaging of tissues inside or between the eyes.

In zebrafish, multiple populations of cells produce pigments that ultimately restrict the optical accessibility of tissues^5^,^6^. The three main kinds of pigment cells (or chromatophores) are the melanophores (black appearance), iridophores (silvery or blue) and xanthophores (yellow) (Fig. 1a). In addition to these three populations, which in zebrafish larvae derive from neural crest cells, there is another population of pigment cells forming the retinal pigment epithelium (RPE), which originates from optic lobe neuroepithelial cells (Fig. 1a)^7^. Common strategies to reduce zebrafish skin pigmentation can be grouped in two categories: 1) use of chemical compounds interfering with the synthesis of melanin, the most popular one being 1-phenyl-2-thiourea (PTU)^8^; 2) use of pigmentation mutants in which genes controlling either pigment formation, chromatophore formation or interactions between chromatophores have been inactivated^4^,^6^,^9^. The first strategy, despite being straightforward (i.e., embryos can be simply raised in medium containing 200 μM PTU), is associated with toxic side effects that impair morphogenesis, behaviour and survival. For example, PTU has been shown to interfere not only with tyrosinase (the enzyme that catalyses the production of melanin)^10^,^11^, but also with other enzymes, such as thyroid peroxidase^12^ and dopamine beta-hydroxylase^13^. Therefore, the poor selectivity of this drug results in severe consequences including reduced thyroid hormone synthesis^14^, decreased eye size^12^, abnormal cranial neural crest and extraocular muscle development^15^, impaired retinal light adaptation^16^, as well as anxiety^17^ and reduced mobility, hatching and survival^8^ (see also Results and Discussion). In contrast, the second strategy is considerably less disruptive since it takes advantage of viable mutations affecting the function of genes expressed in specific subsets of cells where they are involved in defined processes of pigment production^5^. Since the formation of each pigment type is controlled independently of the others, the combination of different mutations is required to produce fully transparent zebrafish. This strategy has been previously implemented to generate the double mutant *casper*, which lacks all melanophores and iridophores^4^. Here, we further develop this strategy to generate a fully optically clear combinatorial mutant (*crystal*) that not only lacks all melanophores and iridophores, but also has a non-pigmented RPE. This particular feature makes *crystal* larvae especially suited for imaging tissues inside or between the eyes while avoiding the use of chemical pigmentation blockers. Importantly, *crystal* mutants show no behavioural or viability deficits as compared to wild type animals. We also validate the *in vivo* application of this novel mutant by performing whole-brain light-sheet imaging and two-photon functional recordings of neural activity in the retinae of *crystal* larvae.

## Results and Discussion

### Generation of *crystal*: a fully transparent zebrafish for *in vivo* imaging

**Figure 1:**
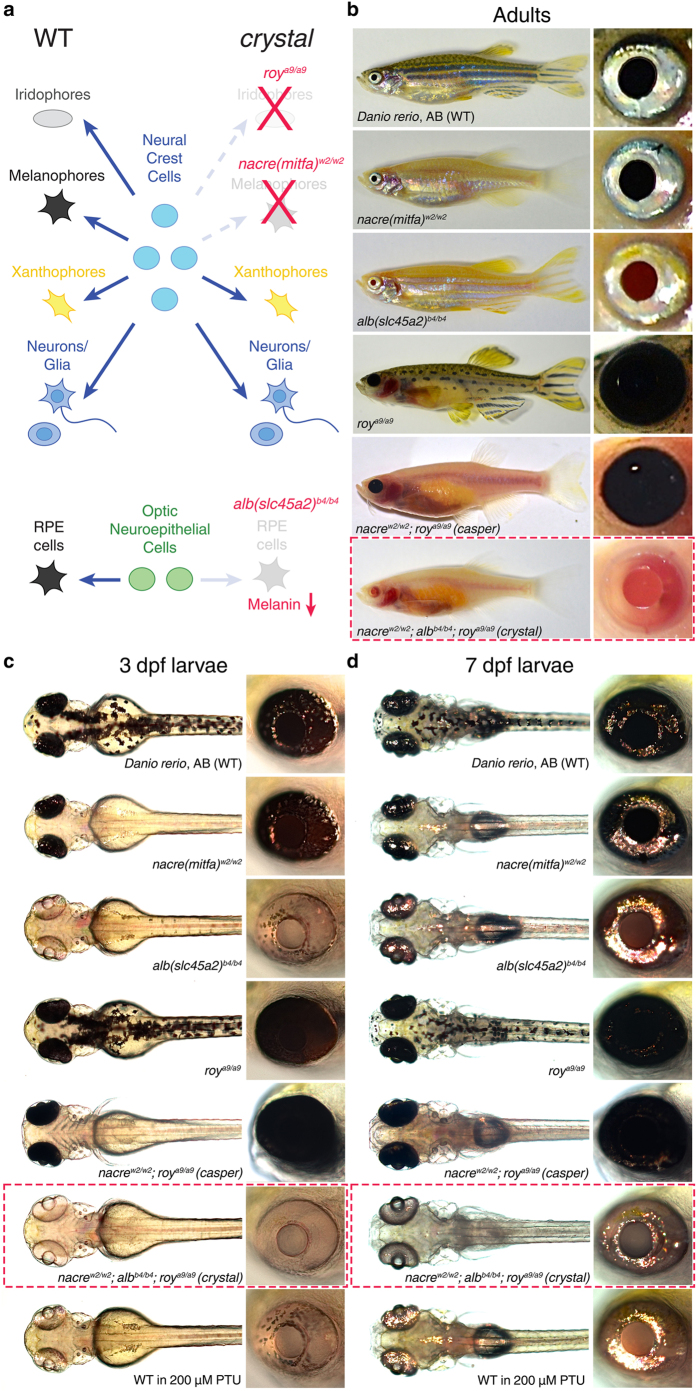
Generation of *crystal*, a fully transparent combinatorial pigmentation mutant. (**a**) Left: schematic diagram of the main populations of pigment cells in wild type (WT) zebrafish. Centre: cell lineages generating the different populations of pigment cells. Right: mutations affecting genes controlling either pigment cell formation (*nacre*^*w2/w*^2 and *roy*^*a9/a9*^) or melanin production (*alb*^*b4/b4*^) used to generate the *crystal* mutant. RPE, retinal pigment epithelium. (**b–d**) Pigmentation phenotypes of wild type, single mutant, *casper* and *crystal* zebrafish at adult (>3 month old, (**b**)), embryonic (3 dpf, (**c**)) and larval (7 dpf, (**d**)) stages. Red dashed boxes indicate *crystal* mutants. Insets on the right display eye pigmentation phenotypes. 3 dpf and 7 dpf zebrafish treated with 200 μM PTU are shown at the bottom of (**c**,**d**). Note that the optical transparency of *crystal* fish is higher than that of wild type, single mutant, *casper* and PTU-treated fish.

To generate optically clear zebrafish lacking the vast majority of skin pigmentation without using chemical compounds inhibiting pigment formation, we undertook a combinatorial genetic approach. Three previously described viable mutations affecting different populations of chromatophores (Fig. 1a) were selected and combined through crossbreeding (Fig. 1b): 1) the *nacre*^*w2/w2*^ mutant lacks all melanophores due to a mutation in the *mitfa* gene^9^. Since the *nacre* mutation does not affect the population of cells forming the RPE, this structure is still pigmented in this single mutant. 2) The *albino (alb*^*b4/b4*^) mutant is characterised by a general deficiency in the production of melanin due to a mutation in the *slc45a2* gene^18^. 3) The *roy orbison (roy*^*a9/a9*^) mutant shows a complete lack of iridophores^4^. The gene responsible for this mutant phenotype is currently unknown. The resulting combinatorial triple mutant (*nacre*^*w2/w2*^*;alb*^*b4/b4*^*;roy*^*a9/a9*^; red boxes in Fig. 1b–d), which we name *crystal*, lacks the vast majority of dark and reflective pigments normally present at the cutaneous level and, as a consequence, appears optically clear. The internal organs are clearly visible in adult (>3 months old) *crystal* mutants as opposed to wild type and single mutant fish (Fig. 1b). Compared to the previously described double mutant *casper (nacre*^*w2/w2*^*;roy*^*a9/a9*^)^4^, where internal organs are also visible, the triple mutant *crystal (nacre*^*w2/w2*^*;alb*^*b4/b4*^*;roy*^*a9/a9*^) lacks melanin in the RPE (Fig. 1b). Consequently, the eyes of *crystal* fish are considerably less pigmented than wild type, single mutant and *casper* fish, therefore resulting easily accessible to optical investigation (see right insets in Fig. 1b–d). The pigmentation phenotype of *crystal* fish is already evident at embryonic (e.g., 3 dpf, Fig. 1c) and larval (e.g., 7 dpf, Fig. 1d) stages. Importantly, the optical clarity of *crystal* larvae is even superior to that of larvae treated with 200 μM PTU (Fig. 1c,d), since PTU inhibits melanin production but does not interfere with iridophore function^8^. Moreover, unlike PTU-treated animals, adult *crystal* mutants are viable and produce normal numbers of offspring. Heterozygous fish (*nacre*^*w2/*^+;*alb*^*b4/*^+;*roy*^*a9/*^+) do not exhibit any visible phenotype (data not shown). Overall, the *crystal* mutant constitutes a significant improvement in the optical accessibility of both larval and adult zebrafish, even superior to the previously described combinatorial mutant *casper*^4^, which is characterised by black-pigmented eyes.

### *Crystal* zebrafish larvae exhibit normal visual behaviour

**Figure 2:**
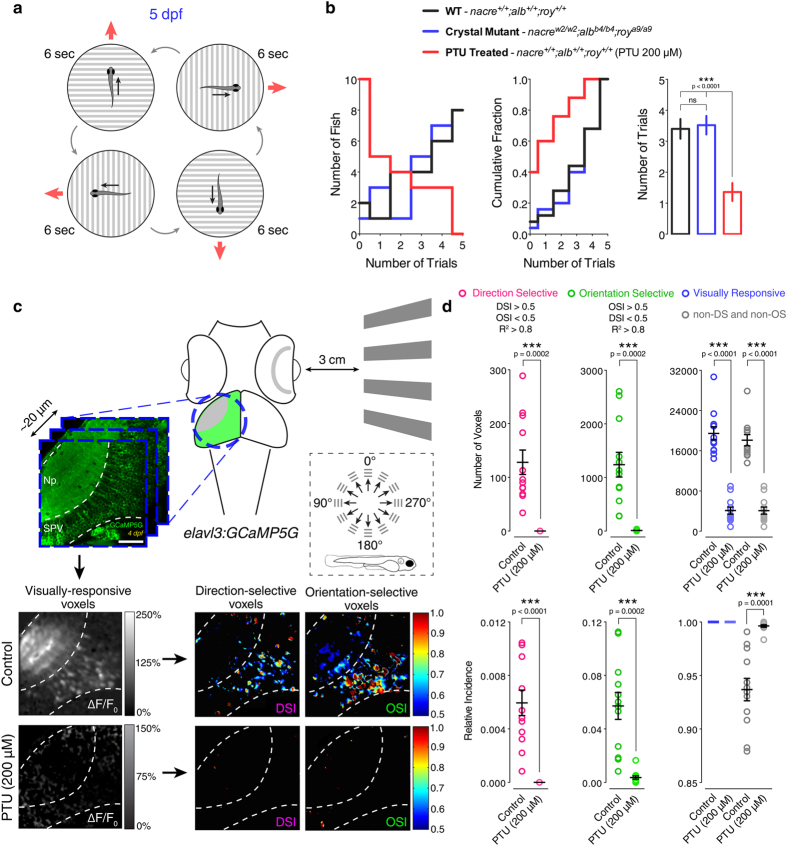
PTU impairs zebrafish behaviour and visual system function. (**a**) Schematic diagram illustrating the optomotor response behavioural assay. Individual 5 dpf larvae were positioned in a petri dish containing Danieau solution. An LCD screen controlled by a computer was used to display black and white square-wave gratings moving in 4 directions (red arrows) at the bottom of the petri dish. Each larva was tested 5 times in total (each trial lasted 6 seconds) and scored according to the trials it responded to (i.e., fish turns and swims in the direction of the moving gratings). (**b**) Quantification of the optomotor response assays for 5 dpf wild type (WT, black), *crystal* (blue) and PTU-treated (red) larvae (n = 25 larvae in each group). The frequency distribution (left), cumulative frequency distribution (centre) and mean ± SEM (right) of number of responsive trials per larva are reported. Note that PTU-treated larvae show a significant decrease in the number of trials they respond to. ns, non-significant; ***p < 0.001; one-way ANOVA with post-hoc Tukey’s HSD test. (**c**) Functional calcium imaging of tectal cells and retinal ganglion cell axons expressing GCaMP5G (green) in 4 dpf *Tg(elavl3:GCaMP5G)* larvae. Distance of right eye from projection screen is 3 cm. Recordings are performed from 3 Z-planes (approximately 20 μm total volume thickness) in the contralateral optic tectum at 4 Hz image acquisition rate. Dashed box shows the angles of moving bars relative to zebrafish larva orientation. Mean ΔF/F_0_ images of calcium recordings in untreated (control) and PTU-treated larvae followed by mapping of direction-selective (DS) and orientation-selective (OS) voxels are displayed. Np, neuropil; SPV, stratum periventriculare; DSI, direction selectivity index; OSI, orientation selectivity index. Scale bar is 40 μm. (**d**) Average number (top) and relative frequency (bottom) of DS, OS, visually responsive and non-DS/non-OS voxels per Z-plane in control and PTU-treated 4 dpf larvae (n = 12 larvae in each group). Criteria used to identify DS and OS voxels are reported at the top. Note the dramatic reduction in visually responsive, DS and OS voxels following 200 μM PTU treatment. Error bars are ± SEM. ***p < 0.001, unpaired two-tailed Student’s t test.

An ideal system for *in vivo* imaging has to be characterised not only by optical clarity but also by normal functional and behavioural viability. The use of PTU to quickly obtain transparent zebrafish larvae is widespread across the research community. However, numerous studies reported severe morphological and behavioural side effects caused by the toxicity of PTU treatment (see Introduction). We thus wanted to compare the behavioural viability of *crystal* larvae to wild type larvae and to larvae raised in medium containing 200 μM PTU. As a representative visually guided behaviour we tested the ability of 5 dpf larvae to perform the optomotor response. During optomotor response assays, freely swimming zebrafish larvae respond to whole-field moving stimuli (e.g., dark and light bars) by swimming in the same direction of stimulus motion (Fig. 2a)^16^. They do so to compensate for the optic flow-induced perception of apparent involuntary displacement, and therefore regain the desired course of locomotion. Individual larvae were tested five times in total and scored according to the number of trials they responded to (i.e., fish turns and swims in the direction of the moving stimulus). Notably, *crystal* larvae exhibited a response rate equivalent to wild type larvae as opposed to PTU-treated larvae, which instead showed a dramatic behavioural impairment (Fig. 2b).

Given the observed behavioural consequences of PTU treatment, we further investigated whether PTU has any effect on zebrafish visual system function. We did so by recording visually induced neural responses in the optic tectum, which in zebrafish is the main retinorecipient brain area. The zebrafish optic tectum receives inputs from all functional types of retinal ganglion cells^19^, the sole output neurons of the retina, including ganglion cells tuned to stimulus direction of motion (direction-selective cells) or stimulus orientation (orientation-selective cells)^20^. Using a transgenic fish line where the genetically encoded calcium indicator GCaMP5G is expressed pan-neuronally^21^, we analysed visual responses to moving dark and light bars in both the tectal neuropil and periventricular neurons through *in vivo* calcium imaging, as previously described (Fig. 2c and Supplementary Video 1)^22^,^23^. Compared to untreated *Tg(elavl3:GCaMP5G)* larvae, 4 dpf PTU-treated larvae (treatment from 1 dpf to 3 dpf) showed a large decrease in visual responses with direction- and orientation-selective neural responses being absent or severely impaired, respectively (Fig. 2c). From these data, we conclude that PTU can cause deleterious consequences on both zebrafish behaviour and neural function, and should be therefore avoided whenever these two biological processes are under investigation. Since PTU is a highly non-selective drug (see Introduction) and is generally applied at the whole-animal level, it is difficult to attribute any of the detrimental effects we observed to a specific biochemical pathway.

### Whole-brain light-sheet imaging of *crystal* larvae

**Figure 3:**
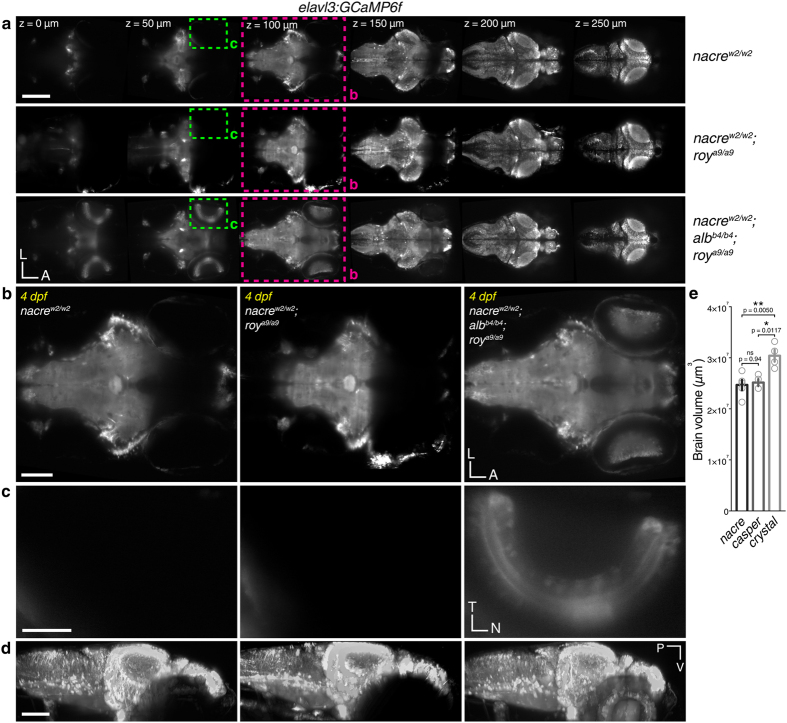
Improved optical accessibility of zebrafish larvae in whole-brain light-sheet imaging using *crystal.* (**a**) Volumetric imaging of the larval zebrafish brain with light-sheet microscopy in 4 dpf *nacre* (top), *casper* (middle) and *crystal* (bottom) *Tg(elavl3:GCaMP6f)* larvae (n = 4–5 larvae in each group). Six different volume sections per larva out of 450 (total xyz volume of 798 × 623 × 283 μm^3^) are displayed. L, left; A, anterior. Scale bar is 200 μm. (**b**) Single volume sections of the brain (100 μm Z plane depth) in *nacre* (left), *casper* (middle) and *crystal* (right) larvae. Note the dark region between the eyes of *nacre* and *casper* larvae due to the excitation light being absorbed or reflected by pigments present on the surface of the eyes. L, left; A, anterior. Scale bar is 100 μm. (**c**) Insets showing the labelling of amacrine and ganglion cells in the left retina of the *crystal* larva (right) compared to *nacre* (left) and *casper* (middle) larvae, where no GCaMP6f fluorescence is detected in the eyes. T, temporal; N, nasal. Scale bar is 50 μm. (**d**) 3D reconstructions of the brain (lateral view) of *nacre* (left), *casper* (middle) and *crystal* (right) *Tg(elavl3:GCaMP6f)* larvae shown in (**a**). Note the improved optical accessibility (~18% of brain volume) allowed by *crystal*. P, posterior; V, ventral. Scale bar is 100 μm. (**e**) Average imaged brain volume in *nacre, casper* and *crystal* 4 dpf *Tg(elavl3:GCaMP6f)* larvae. Error bars are ± SEM. ns, non-significant; *p < 0.05; **p < 0.01; one-way ANOVA with post-hoc Tukey’s HSD test.

We next aimed to validate the *in vivo* application of *crystal* larvae by performing whole-brain light-sheet imaging^24^. Light-sheet microscopy has recently experienced a series of significant technological advancements and is generally used to study nervous system activity and development with cellular resolution at the whole-animal level^21^,^25^,^26^,^27^. In typical zebrafish light-sheet preparations, the excitation light is provided laterally and fluorophore emission light is collected through an objective positioned orthogonally to the illumination plane. However, due to the strong pigmentation of the eyes, imaging of areas inside or between these structures is normally problematic. We therefore compared the optical accessibility of the nervous system between *crystal, nacre* and *casper* larvae at 4 dpf using the pan-neuronal *Tg(elavl3:GCaMP6f)* line^28^ (Fig. 3a and Supplementary Video 2). In our setup, the sheet of laser light (488 nm) is generated by two objectives positioned on the lateral sides of the larva. In *crystal* larvae, the excitation light can easily reach deep (i.e., ventral) regions of the brain (Fig. 3b) as well as the retinae (Fig. 3c) without being absorbed or reflected by pigments normally present on the surface of the eyes, like in *nacre* or *casper* larvae (note the dark regions in the left and middle panels of Fig. 3b,c). Therefore, *crystal* allows a fully unobstructed optical access of the larval zebrafish brain in its entirety (i.e., including the eyes), as opposed to *nacre* and *casper* mutants where a substantial portion of the nervous system (~18%) is not accessible through standard light-sheet imaging (Fig. 3a,d,e; mean volume ± SEM, *crystal* 3.04 ± 0.12 × 10^7^ μm^3^, n = 4; *casper* 2.51 ± 0.07 × 10^7^ μm^3^, n = 4; *nacre* 2.47 ± 0.10 × 10^7^ μm^3^, n = 5; p = 0.0040, F_2,10_ = 10.09, one-way ANOVA with post-hoc Tukey’s HSD test). Even though the optical accessibility of crystal larvae through light-sheet imaging is significantly higher than *nacre* and *casper* mutants, it is noteworthy that the spatial definition of brain regions between the eyes appears lower than other regions of the brain, likely due to scattering of excitation light by the lenses. Despite this fact, the light-sheet signal detected from these regions in *crystal* mutants is still many-fold higher than in the other animal groups (Fig. 3a,b).

### Two-photon calcium imaging in the intact retina using *crystal*

**Figure 4:**
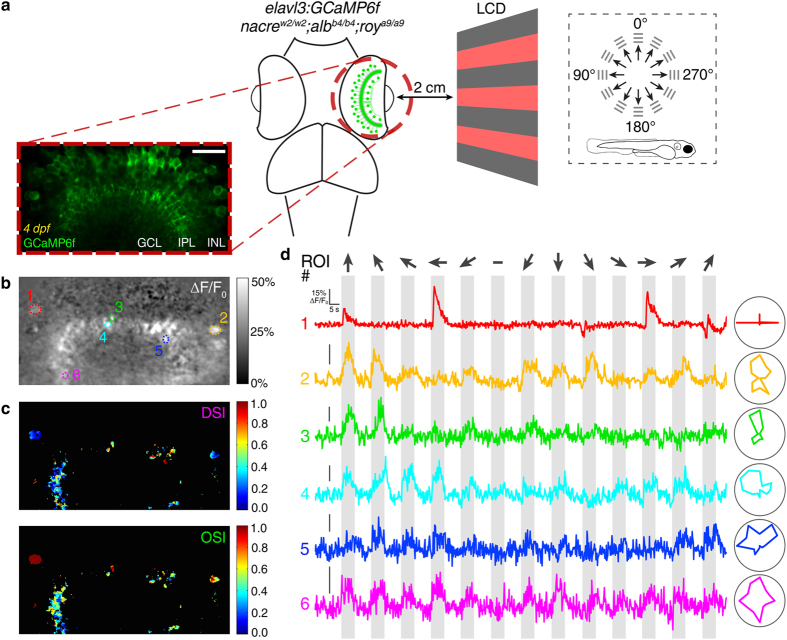
Calcium imaging of visually evoked neural activity in the retina using *crystal.* (**a**) Two-photon functional calcium imaging of amacrine cells and ganglion cell dendrites expressing GCaMP6f (green) in 4 dpf *crystal Tg(elavl3:GCaMP6f)* larvae (n = 8 larvae). Distance of the eye from LCD screen is 2 cm. Recordings are performed from 2–4 Z-planes (approximately 20 μm total volume thickness) at 4 Hz image acquisition rate. Dashed box shows the angles of moving gratings relative to zebrafish larva orientation. INL, inner nuclear layer; GCL, ganglion cell layer; IPL, inner plexiform layer. Scale bar is 20 μm. (**b,c**) Mean ΔF/F_0_ image of a representative calcium recording (**b**) followed by voxel-wise analysis of direction and orientation selectivity of visual responses (**c**). DSI, direction selectivity index; OSI, orientation selectivity index. (**d**) ΔF/F_0_ calcium traces during a representative tuning experiment from the 6 selected regions of interest (ROIs) shown in (**b**). Polar plots showing the tuning profiles (i.e., integral ΔF/F_0_ responses to different stimuli) of individual ROIs are reported on the right. Stimulus epochs are shown in grey. Dark arrows indicate the different directions of gratings motion. The blank-screen null condition is indicated by a ‘-’ sign.

To further assess the *in vivo* application of *crystal* larvae we performed two-photon functional imaging of neural activity in the retina (Fig. 4a), a brain region that is not optically accessible in wild type, single pigmentation mutant or *casper* larvae (Fig. 1) unless embryos are raised in medium containing PTU^29^,^30^. The retina is a sensory neural circuit formed by multiple classes of excitatory cells (photoreceptors, bipolar and ganglion cells) and inhibitory cells (horizontal and amacrine cells)^31^. Its primary function is to detect and process light stimuli and, subsequently, send the processed visual information to higher brain areas through different types of functionally specialised ganglion cells^32^. To record visual responses in the larval retina through calcium imaging, 4 dpf *Tg(elavl3:GCaMP6f) crystal* larvae were immobilised in 2% low melting point agarose with one eye facing an LCD screen where square-wave gratings moving in 12 different directions were displayed (n = 8 larvae). Visually evoked calcium transients were recorded from amacrine and ganglion cells at 4 Hz using near-infrared (930 nm) two-photon laser excitation (Supplementary Video 3). Voxel-wise analysis was then used to identify visually responsive voxels (Fig. 4b) and quantify direction and orientation selectivity of visual responses at subcellular resolution (0.248 × 0.248 μm voxel XY size, Fig. 4c) as previously described^22^,^33^. ΔF/F_0_ calcium traces from 6 selected regions of interest (ROIs, Fig. 4b) are displayed as examples (Fig. 4d). Interestingly, not only we could record stimulus-locked responses, but also we observed the presence of direction-selective (direction selectivity index (DSI) > 0.5, ROI# 4 and 5) and orientation-selective (orientation selectivity index (OSI) > 0.5, ROI# 1–3) responses (Fig. 4c,d), indicating that the retina is functional in *crystal* larvae. A previous study has reported that adult zebrafish mutants with hypopigmented eyes show deficits in performing visual escape assays under defined luminance conditions^34^. Thus, even though our visual behaviour assays demonstrated that *crystal* larvae perform the optomotor response as well as wild type larvae (Fig. 2a,b), we cannot exclude that *crystal* mutants might show abnormal retinal responses under certain luminance conditions. However, given the deleterious effects caused by PTU treatment (Fig. 2c,d; see Introduction), the *crystal* mutant represents, to the best of our knowledge, the most viable strategy to perform *in vivo* functional imaging in the intact retina of larval zebrafish.

## Conclusions

In this study, we generated a viable, optically transparent combinatorial pigmentation mutant zebrafish, named *crystal*, which constitutes an ideal tool for completely unobstructed *in vivo* imaging of biological phenomena. More specifically, compared to *casper* mutants^4^, *crystal* fish are superior in terms of optical transparency when imaging inside or between the eyes. We validated the viability and *in vivo* application of *crystal* larvae through the optomotor response assay, whole-brain light-sheet imaging and two-photon functional imaging of neural activity in the intact retina. Importantly, *crystal* larvae show a higher viability than larvae treated with the chemical pigmentation blocker PTU. We envisage that *crystal* will be an invaluable tool for other *in vivo* applications, such as one-photon^21^ or two-photon^27^ volumetric calcium imaging of neural activity across the entire brain in semi-restrained behaving^35^ or paralysed fictively swimming^36^ zebrafish larvae, as well as to study the wiring, function and plasticity of neural circuits in normally highly pigmented, optically inaccessible structures like the eyes^30^,^37^,^38^.

## Methods

### Animals

Zebrafish were maintained at 28.5 °C on a 14 hr ON/10 hr OFF light cycle in Danieau solution [58 mM NaCl, 0.7 mM KCl, 0.4 mM MgSO_4_, 0.6 mM Ca(NO_3_)_2_ 5.0 mM HEPES, pH 7.6]. The combinatorial *crystal* mutant (*alb*^*b4/b4*^;*nacre*^*w2/w2*^;*roy*^*a9/a9*^) was generated by sequentially crossbreeding three different single mutant strains, namely *nacre*^*w2/w2*^ (ref. 9), *roy*^*a9/a9*^ (ref. 4) and *alb*^*b4/b4*^ (ref. 18) mutants. Importantly, both larvae and adult *crystal* zebrafish are viable and do not display visible morphological, functional or behavioural abnormalities other than the pigmentation phenotype. The *casper* mutant was obtained by crossing *nacre*^*w2/w2*^ and *roy*^*a9/a9*^ mutants, as previously described^4^. The *AB* line was used to obtain wild type zebrafish. Transgenic lines used in this study include *Tg(elavl3:GCaMP5G)*^21^ and *Tg(elavl3:GCaMP6f)*^28^. Confocal functional imaging experiments were performed in the *nacre* mutant. Light-sheet imaging experiments were performed in *nacre, casper* and *crystal* mutants. Two-photon functional imaging experiments were performed in *crystal* larvae. To treat zebrafish larvae with PTU we followed standard procedures^8^, specifically larvae were raised in 200 μM PTU (Sigma) in Danieau solution from 24 hours post fertilisation (hpf). *Tg*(*elavl3:GCaMP5G*) larvae used for confocal functional imaging of visually evoked neural activity in the optic tectum were treated with 200 μM PTU from 24 hpf to 3 dpf and imaged at 4 dpf. This work was approved by the local Animal Welfare and Ethical Review Body (King’s College London), and was carried out in accordance with the *Animals (Scientific Procedures*) *Act 1986*, under license from the United Kingdom Home Office (PPL70/8057).

### Imaging

#### Whole-animal *in vivo* microscopy

Whole-animal images of adult zebrafish were taken with a Nikon D7000 digital SLR camera equipped with a Sigma 150 mm macro lens. Adult zebrafish were anesthetized with 0.2% tricaine (MS222, Sigma) in fish facility water and placed in a 90 mm petri dish containing fish facility water. Imaging of larvae was performed using a ZEISS Axioskop microscope connected to EXi Blue CCD cameras (Retiga) and Volocity acquisition software (PerkinElmer). Larval zebrafish were anesthetized with 0.02% tricaine in Danieau solution and immobilized in 1% low melting point agarose (Sigma) on glass slides.

#### Confocal calcium imaging

Imaging was performed using a ZEISS LSM 710 confocal microscope equipped with a spectral detection scan head and a 20X/1.0 NA water-immersion objective (Carl Zeiss). Functional time-series of visually evoked calcium responses in retinal ganglion cells (RGCs) were acquired at a rate of 4.1 Hz and 0.415 × 0.415 μm resolution (256 × 256 pixels), and 1 AU pinhole aperture. Excitation light was provided by a 488 nm multi-line laser. Non-anaesthetised *Tg(elavl3:GCaMP5G)* larvae were immobilized in 2% low melting point agarose (Sigma) prepared in Danieau solution and mounted dorsal side up on a raised glass platform that was placed in a custom-made Danieau-filled chamber. The agarose was sufficient to restrain the larvae so that anaesthesia was not required. Imaging was performed in the afternoon (1–8 pm).

#### Light-sheet imaging

Whole-brain light-sheet imaging was performed using a ZEISS Lightsheet Z.1 microscope equipped with two 10X/0.2 NA illumination objectives and one 20X/1.0 NA water-immersion detection objective (Carl Zeiss). 488 nm laser excitation light was used to elicit GCaMP6f fluorescence and a 505–545 BP filter was used for emitted light detection. The pivot scanner (Carl Zeiss) was used to deliver homogeneous illumination and, therefore, avoid shadows along the illumination axis. The thickness of the light sheet was 5.39 μm at the centre and 10.8 μm at the edges of the field of view. Exposure time was 29.97 ms. The size of volumetric images was 623 × 798 × 283 μm^3^ (1500 × 1920 × 490 pixels) with a resolution of 0.415 × 0.415 × 0.631 μm. 4 dpf *nacre, casper* and *crystal Tg(elavl3:GCaMP6f)* larvae were first paralysed for 10–15 minutes in α-bungarotoxin (1 mg/ml; Biotium) prepared in Danieau solution. Subsequently, larvae were immobilized in 2% low melting point agarose (Sigma) and placed inside a glass capillary (20 μl volume, 701904; Brand). We subsequently extruded the section of the agarose cylinder containing the head of the larva from the capillary, and oriented the larvae so that the dorsal side of the head was facing the detection objective and the eyes were facing the two illumination objectives. Whole-brain light-sheet imaging of *casper* mutant larvae was performed using a custom-made light-sheet microscope built by Dr Martin Meyer (King’s College London) and equipped with a 20X/1.0 NA water-immersion XLUMPlanFLN detection objective (Olympus).

#### Two-photon calcium imaging

Two-photon functional imaging in the retina was performed using a Nikon A1R MP microscope equipped with a 4-channel GaAsP NDD and an Apochromat 25X/1.1 NA water-immersion objective (Nikon). Excitation was provided by a Chameleon Ultra II Mode-locked titanium-sapphire laser (Coherent) tuned to 930 nm. Time-series of visually evoked calcium responses were acquired at a rate of 4 Hz and 0.248 × 0.248 μm resolution (512 × 256 pixels). Following activation of the laser scanning, we waited 60 seconds before starting the visual stimulation to ensure the retina adapted to the background light level caused by the multi-photon laser. 4 dpf *crystal Tg(elavl3:GCaMP6f)* larvae were first paralysed for 10–15 minutes in α-bungarotoxin (1 mg/ml; Biotium) prepared in Danieau solution. Subsequently, larvae were immobilized in 2% low melting point agarose (Sigma) and mounted on a raised custom-made glass platform with the dorsal side up (45° angle tilt) and one eye facing an LCD screen (see Visual stimulation) that was placed underneath a custom-made Danieau-filled chamber. Imaging was performed in the afternoon (1–8 pm).

### Visual stimulation

#### Moving bars in confocal preparation

Moving bars stimuli were generated as previously described^22^,^33^. A diffusion filter (3026, Rosco) was bonded to one side of the chamber to serve as a projection screen. The agarose in front of the eye facing the projection screen was removed, allowing an unobstructed view of the projected image on the side of the chamber. Larvae were positioned 3 cm away from the screen and the projected image filled a visual field of ~97° × 63°. Visual stimuli consisted of light (56 cd/m^2^) or dark bars (8 cd/m^2^) (175% and 25% of mean luminance, respectively) on a mean grey background (32 cd/m^2^). As no qualitative differences between light and dark bars were noted, data obtained using the two stimuli were combined. Each bar was 10° in width moving at a speed of 20°/s and separated from the preceding bar by 30°, enabling more than one bar on the screen at any one time. The long axes of the bars were orthogonal to the direction of motion. Each of the 12 directions of motion was presented once (3 seconds) in a pseudo-random order unique to each slice in every animal imaged. Each inter-epoch interval was 10 seconds to enable GCaMP5G signals to return to baseline. A blank-screen null condition of 2 seconds was also interleaved. Visual experiments were generated and controlled using custom-written Labview and MATLAB code (MathWorks), implemented on a ViSaGe stimulus presenter (Cambridge Research Systems) and delivered via a DLP Pico Projector (Optoma).

#### Moving gratings in two-photon preparation

Moving gratings stimuli in the two-photon preparation were generated and controlled using PsychoPy^39^, and delivered through an LCD screen (SKD5VA-3, GoodWell Technology) positioned underneath a custom-made Perspex chamber. A long-pass red glass filter (FGL610, Thorlabs) was positioned between the LCD screen and the chamber to allow for simultaneous imaging and visual stimulation. Larvae were positioned 2 cm away from the screen and the image on the LCD screen filled a visual field of ~140° × 100° (mean background luminance 30.4 cd/m^2^). Visual stimuli consisted of square-wave gratings (100% contrast, spatial frequency 1.66 cycles/cm, temporal frequency 1 cycles/s). Each grating bar was 8.5° in width and the long axes of the bars were orthogonal to the direction of motion. Each of the 12 directions of motion was presented once (6 seconds) with and inter-epoch interval of 10 seconds to enable GCaMP6f signals to return to baseline. A blank-screen null condition of 6 seconds was also interleaved. TTL triggers (0-5-0 Volts) to record epoch time events where generated through a LabJack USB DAQ device (U3-LV, LabJack Corporation). Following activation of the laser scanning, we waited 60 seconds before starting the visual stimulation to ensure the retina adapted to the background light level caused by the multi-photon laser.

#### Optomotor response assay

Individual 5 dpf larvae were positioned in a 35 mm petri dish containing Danieau solution. The LCD screen of an iPhone 5 (Apple) controlled by a MacBook Pro (Apple) through Duet Display (Kairos Technologies) was used to display black and white square-wave gratings (85% contrast, spatial frequency 0.33 cycles/mm, temporal frequency 3.5 cycles/s) moving in 4 directions (90° angular distance) at the bottom of the petri dish. Visual stimuli were generated in Keynote (Apple). Each larva was tested 5 times in total (each trial lasted 6 s followed by 10 s of static gratings) and scored according to the trials it responded to (i.e., fish turns and swims in the direction of the moving gratings). The behaviour of larvae was visually monitored using an M165 FC stereomicroscope (Leica).

### Analysis

#### Functional Analyses

*In vivo* calcium imaging data were analysed as previously described^22^,^33^. In summary, functional time-series were processed before analysis as follows: time-series images from each experiment were corrected for motion with a rigid-body algorithm (SPM12; http://www.fil.ion.ucl.ac.uk/spm/), median filtered with a kernel size of 1 voxel to remove dark and shot noise, and spatially smoothed with a 2D Gaussian kernel = 2 voxels to improve signal-to-noise. A baseline (B) that corrects for low-frequency drifts was determined using a cubic-spline algorithm extrapolating between knots averaged from 5 s of the inter-epoch interval data. Both relative signal intensity changes (ΔF = F-B; where F = raw fluorescence) and normalised signal intensity changes [%ΔF/F_0_ = (F-B)/B] were calculated at each voxel. ΔF was used for population functional data (voxel-wise analysis), whereas %ΔF/F_0_ was used for manually defined regions of interest (ROIs). For each voxel or ROI the integral response over the epoch-interval was calculated to provide a single response metric of each presented direction of stimulus motion. The integral within each epoch window is a summary metric more resistant to saturation effects of the calcium probe than maximum signal change. A threshold for each voxel within an acquisition image sequence was determined from the variance of ΔF changes during the inter-epoch intervals and null condition, threshold = 5 × SDs. All voxels that were supra-threshold within at least two visual presentation epochs were regarded as visually responsive and subjected to further characterization.

To analyse the direction and orientation selectivity of visually responsive voxels direction- and orientation-selective indices (DSI and OSI)^40^, based on fitted von-Mises or Gaussian profiles^41^, were calculated together with an estimate for their goodness of fit, R^2^. The DSI was defined as (R_pref_−R_null_)/(R_pref_ + R_null_), where R_pref_, the response to the preferred direction, was the integral response over the preferred direction epoch-interval. R_null_ was similarly calculated as the integral response evoked by the direction opposite to the preferred direction. The OSI was defined as (R_pref_−R_orth_)/(R_pref_ + R_orth_), where R_pref_, the response to the preferred orientation, was the integral response over the preferred orientation epoch-interval. R_orth_ was similarly calculated as the integral response evoked by the orthogonal orientation. To minimize cross talk and over-fitting associated with DSI and OSI metrics, a stringent approach was undertaken. For a voxel to be regarded as direction-selective (DS) or orientation-selective (OS), mutually exclusive criteria were used: DS if DSI > 0.5 and OSI < 0.5; and OS if OSI > 0.5 and DSI < 0.5. In both cases, the goodness of fit (R_2_) for DSI and OSI, respectively, had to be >0.8; thus, the fitted curves explained at least 80% of the integral responses. A single von-Mises distribution was used to fit responses of DS voxels and estimate their preferred direction of motion angle from the centre of the fitted curve. The sum of two von-Mises (180° angular distance apart) was used to fit responses of OS voxels and estimate their preferred orientation of motion angles from the centres of the fitted curves. Circular variance was also calculated for comparison as an alternative metric of orientation selectivity (Circular variance < 0.4)^41^.

#### Morphological Analyses

To determine the brain volume imaged in 4 dpf *nacre, casper* and *crystal Tg(elavl3:GCaMP6f)* larvae, we calculated the number of GCaMP6f^+^ voxels in each volumetric image by applying the adjust>threshold function followed by the analyse>histogram>list command in ImageJ^42^. Subsequently, the obtained values were multiplied by the volume of a single voxel (0.415 × 0.415 × 0.631 μm^3^ = 1.086 × 10^−1^ μm^3^).

#### Statistical Analyses

Statistical test results are reported in Figures and Figure legends. Statistical analyses and tests were carried out using Prism 6 (GraphPad) or MATLAB R2014b (MathWorks). Before performing statistical tests, descriptive statistics (e.g., normality tests to see whether values come from a Gaussian distribution or F-test to compare variances) were used to choose the appropriate statistical test (reported in Figure legends together with test results). The criterion for statistical significance was set at p < 0.05.

## Additional Information

**How to cite this article**: Antinucci, P. and Hindges, R. A *crystal*-clear zebrafish for *in vivo* imaging. *Sci. Rep.* **6**, 29490; doi: 10.1038/srep29490 (2016).

## Supplementary Material

Supplementary Information

Supplementary Video 1

Supplementary Video 2

Supplementary Video 3
