# Supplementary material for: A crystal-clear zebrafish for in vivo imaging
Source: Sci Rep. 2016 Jul 6;6:29490. doi: 10.1038/srep29490 (PMC4933947; doi:10.1038/srep29490)
Supplement: Supplementary Information [file srep29490-s1.pdf]

Supplementary Information

A *crystal*-clear zebrafish for *in vivo* imaging

Paride Antinucci[1]* and Robert Hindges[1]*

[1]MRC Centre for Developmental Neurobiology
King's College London
Guy's Campus
London SE1 1UL
UK

* *Correspondence:* paride.antinucci@kcl.ac.uk, robert.hindges@kcl.ac.uk

Supplementary Video Titles and Legends

Supplementary Video 1. Functional imaging of neural activity in the optic tectum of control and PTU-treated zebrafish larvae

Time-lapse functional calcium imaging of visually evoked activity in the optic tectum of representative control (top) and PTU-treated (bottom) 4 dpf *nacre Tg(elavl3:GCaMP5G)* larvae. The movies encompass entire tuning experiments during which bars moving in 12 directions plus a blank screen null stimulus are presented to the right eye of immobilised zebrafish larvae. Unprocessed GCaMP5G responses are shown on the left, whereas $\Delta F/F_0$ calcium responses are reported on the right. The direction of moving stimuli (°) is displayed when bars are projected onto the screen. Time is given in min:sec. The acquisition rate is 4 Hz, and the movie speed is approximately 5x. Anterior is up, posterior is down.

Supplementary Video 2. Whole-brain light-sheet imaging in *nacre* and *crystal* mutant zebrafish

Z-stacks acquired through light-sheet microscopy showing the brain of representative *nacre* (left) and *crystal* (right) 4 dpf *Tg(elavl3:GCaMP6f)* larvae. Relative Z-axis depth is reported in μm (total volume of 623 × 798 × 283 μm^3 with 0.415 × 0.415 × 0.631 μm resolution). Anterior is up, posterior is down.

Supplementary Video 3. Functional imaging of neural activity in the retina of *crystal* zebrafish

Time-lapse two-photon calcium imaging of visually evoked activity in amacrine and ganglion cells in the retina of a representative 4 dpf *crystal Tg(elavl3:GCaMP6f)* larva. The movie encompasses an entire tuning experiment during which gratings moving in 12 directions plus a null stimulus are presented to one eye of the immobilised zebrafish larva. GCaMP6f fluorescence responses are shown at the top, whereas $\Delta F/F_0$ calcium

responses are reported at the bottom. The direction of moving stimuli (°) is reported when gratings are displayed on the LCD screen. Time is given in min:sec. The image acquisition rate is 4 Hz, and the movie speed is approximately 5x.
